# A genetically encoded, high-signal-to-noise maltose sensor

**DOI:** 10.1002/prot.23118

**Published:** 2011-10-12

**Authors:** Jonathan S Marvin, Eric R Schreiter, Ileabett M Echevarría, Loren L Looger

**Affiliations:** 1 Janelia Farm Research Campus, Howard Hughes Medical InstituteAshburn, Virginia 20147; 2 Department of Chemistry, University of Puerto RicoRío Piedras Campus, San Juan, Puerto Rico 00931; 3 Department of Biochemistry, University of Puerto Rico, Medical Sciences CampusSan Juan, Puerto Rico 00936

**Keywords:** periplasmic binding protein, genetically encoded indicator, sensor, maltose, fluorescent protein

## Abstract

We describe the generation of a family of high-signal-to-noise single-wavelength genetically encoded indicators for maltose. This was achieved by insertion of circularly permuted fluorescent proteins into a bacterial periplasmic binding protein (PBP), *Escherichia coli* maltodextrin-binding protein, resulting in a four-color family of maltose indicators. The sensors were iteratively optimized to have sufficient brightness and maltose-dependent fluorescence increases for imaging, under both one- and two-photon illumination. We demonstrate that maltose affinity of the sensors can be tuned in a fashion largely independent of the fluorescent readout mechanism. Using literature mutations, the binding specificity could be altered to moderate sucrose preference, but with a significant loss of affinity. We use the soluble sensors in individual *E. coli* bacteria to observe rapid maltose transport across the plasma membrane, and membrane fusion versions of the sensors on mammalian cells to visualize the addition of maltose to extracellular media. The PBP superfamily includes scaffolds specific for a number of analytes whose visualization would be critical to the reverse engineering of complex systems such as neural networks, biosynthetic pathways, and signal transduction cascades. We expect the methodology outlined here to be useful in the development of indicators for many such analytes.

## INTRODUCTION

In the last few decades, fluorescent protein (FP)-based sensors that transduce microscopic binding events into macroscopically observable signals have been developed to allow real-time visualization of a variety of biologically relevant molecules (for a comprehensive review of genetically encoded indicators, see Ref.^1^). Protein-based indicators may be expressed in living cells, tissues, and organisms, are easily targeted to specific cellular populations and subcellular locales, and permit imaging with minimally invasive techniques.^2^

In every genetically encoded biosensor, the binding of the analyte to the protein results in a change in the FP properties, permitting detection. One way in which this physical perturbation can be achieved is through a direct interaction with the chromophore of a FP, as is the case in sensors for detection of pH,^3^ halide ions,^4^ redox potential,^5^, ^6^ superoxide,^7^ Ca^2+^ (Ref.^8^), Zn^2+^ (Ref.^9^), and Cu^2+^ (Refs.^9^ and^10^). However, because the FP β-barrel structure provides a single framework for development of chromophore-incorporating binding sites, this strategy is limited in the diversity of analyte that can be detected (e.g., small ions).

One way to diversify the ligand-binding potential of FP-based sensors is to use an exogenous binding domain fused to an FP to allosterically modulate chromophore fluorescence. Proteins that undergo large conformational changes upon ligand binding are good scaffolds in which to establish such allosteric linkages. Calmodulin (CaM) is a textbook example of such a protein: it is in an extended shape in the absence of Ca^2+^ (Ref.^11^) and in a condensed conformation in the presence of Ca^2+^ (Ref.^12^). With a fluorescence resonance energy transfer (FRET) donor and acceptor pair (e.g., cyan and yellow FPs) fused to the N- and C-termini of CaM, the Ca^2+^-dependent conformational change in CaM alters the relative distance and orientation of the two FPs, and thus the FRET efficiency is different in the apo and bound states.^13^ Although FRET-based sensors are ratiometric and thus theoretically more resistant to movement and expression artifacts,^14^ they also have broad spectral footprints, making them less amenable to multianalyte imaging. Donor fluorophores are typically blue-shifted relative to single-FP indicators, which can lead to significant attenuation of excitation light, particularly in two-photon imaging. FRET indicators can also show differential photobleaching of the donor and acceptor FPs, which complicates analysis.^14^ Furthermore, as they require observation of two emission wavelengths, the time frame in which ligand-binding events can be observed is limited by the time required for image acquisition and reflection-cube switching, in the absence of more sophisticated optics. For experiments that occur on time scales in which gene expression, protein degradation, and so forth are expected to influence the observed fluorescence intensity of the sensor, ratiometric sensors can be advantageous. However, for experiments that are performed on much shorter time scales, such as the observation of rapidly fluctuating concentrations of signaling molecules or neurotransmitters, the *in vivo* performance of sensors is more dependent on the signal-to-noise ratio (SNR) and kinetics of the indicator.^15^ It would thus be advantageous to have a generalizable method for increasing the SNRs of FRET sensors. Unfortunately, that does not appear to be straightforward, as even conservative alterations of the binding protein-to-FP linkers may reverse or abolish the ligand-dependent FRET change.^16^, ^17^ Although much work has been done to improve FRET-based genetically encoded calcium indicators,^18^ other FRET-based sensors have seen only incremental improvement at great effort.^17^

An alternative strategy for creating genetically encoded sensors is the allosteric modulation of the fluorescence properties of a single fluorophore, rather than the FRET ratio. Single-FP sensors have a number of advantages: they preserve spectral bandwidth for multianalyte imaging; their saturated states may be nearly as bright as the parental FP; and their ligand-free states may be arbitrarily dim, providing large theoretical fluorescence increases. This allows for much greater changes in fluorescence and thus increased SNRs and greater resistance to photobleaching artifacts.^19^ Furthermore, their smaller size simplifies their fusion to other proteins for organelle localization.

Circularly permuted YFP (cpYFP) has been used as a reporter element in the creation of sensors for H_2_O_2_ (HyPer),^20^ cGMP (FlincG),^21^ and ATP:ADP ratio (Perceval).^22^ Each of these sensors displays at least a doubling of emission intensity upon ligand binding. In each case, only a handful of insertion sites were tested, with little or no optimization of linker composition or length.

More prominently, GCaMP^23^ and improved variants^19^, ^24^, ^25^ have demonstrated that intensity-based sensors possess many advantages over FRET-based indicators for detection of Ca^2+^ transients and neural activity.^19^ GCaMP has cpGFP sandwiched between CaM and the M13 peptide (which binds Ca^2+^-loaded CaM). Efforts to increase the sensitivity of GCaMP for neuronal action potential detection have yielded variants with fluorescence increases (expressed as Δ*F*/*F* = (*F* − *F*_0_)/*F*_0_) greater than 16 in purified protein,^26^ 10 in cell-based assays,^19^ and 2 in awake, behaving worms, flies, and mice.^19^ Crystal structure analysis of GCaMP variants in the Ca^2+^-bound and Ca^2+^-free states^27^, ^28^ has revealed that the sensor mechanism depends on side-chain and solvent modulation of the chromophore tyrosyl protonation state.

It would be valuable to have a general method for developing intensity-based biosensors for a variety of analytes, and the examples listed above are evidence that this should be possible, if the right framework proteins are identified. Periplasmic binding proteins (PBPs) from bacteria undergo dramatic conformational changes upon ligand binding.^29^ X-ray crystal structures of the apo (open) and bound (closed) forms reveal that these proteins have two (typically, although some have more) domains that undergo a large hinge-twist movement relative to each other in a Venus flytrap manner.^30^ This conformational change has been exploited to create a number of FRET-based genetically encoded sensors.^16^, ^31^–^34^ The ligand-binding diversity of the PBP superfamily is immense, making it an attractive source of sensor scaffolds.^30^

Here, we report the creation of a genetically encoded intensity-based sensor from one of the bacterial PBPs in this superfamily. First, we postulated that allosteric coupling of ligand binding to fluorescence would require optimization of two parameters: insertion site and linker composition. We inserted cpGFP into the canonical family member, *E. coli* maltose-binding protein (MBP), at four different locations and performed high-throughput screens to improve the linkers and other sensor elements, resulting in relatively bright sensors with Δ*F*/*F* > 6. Second, we postulated that as the ligand-binding site and chromophore of cpGFP are sterically separated, it should be possible to alter ligand-binding affinity and specificity largely independent of fluorescence. We demonstrate this level of modularity by making point mutations to the binding site that alter maltose affinity and a group of four mutations (previously identified from a genetic selection^35^) that alter the ligand-binding specificity to moderate sucrose preference, both without compromising fluorescence signal change. Furthermore, we demonstrate spectral modularity by changing the residues that comprise the chromophore to yield blue, cyan, green, and yellow sensors. Third, we demonstrate potential *in vivo* applications of the high-affinity maltose sensor by wide-field imaging of maltose uptake in bacteria and addition of extracellular maltose to cultured mammalian cells using two-photon microscopy. Finally, we report the crystal structures of two of the sensors in the maltose-bound state.

We present this work as a proof-of-principle study. With the broad ligand-binding diversity of the PBP family and conserved conformational changes, we expect that other proteins in this family can be converted into bright, high-SNR fluorescence intensity-based sensors if insertion sites are chosen carefully and the residues comprising the linkers are optimized by high-throughput screening.

## MATERIALS AND METHODS

### Cloning

The gene for MBP was cloned by PCR from the *pMalE-p4E* vector (New England Biolabs) into the *pRSET-A* vector (Invitrogen) with *BamHI* and *EcoRI*. The N-terminal periplasmic leader sequence of MBP was excluded, and a *BamHI* site (encoding Gly-Ser) was included at the 5′-end. At the C-terminus, an additional His_6_Gly tag was included after the terminus of MBP (a cloning oversight). An *EcoRI* site was included after the stop codon.

The MBP-cpGFP insertion variants were constructed by overlap PCR using the wild-type MBP sequence and the cpGFP146 variant from GCaMP2.^25^ Detailed sequences are provided in Supporting Information.

For mammalian expression, the MBP165-cpGFP.PPYF.T203V gene was cloned into the pDisplay vector (Invitrogen) at the *BglII* and *PstI* sites.

### Protein expression and purification

Plasmids containing the *MBP-cpGFP* variants were transformed into *E. coli* BL21(DE3) cells (lacking pLysS). Protein expression was induced by growth in liquid autoinduction media supplemented with 100 μg/mL ampicillin at 30°C.^36^ Proteins were purified by immobilized Ni-NTA affinity chromatography.^37^ The MBP-cpGFP proteins were eluted with a 120-mL gradient from 0 to 200 m*M* imidazole.

### Isothermal titration calorimetry

To determine if the MBP-cpGFP variants bound maltose, 15 μ*M* protein (>95% purity on overloaded SDS-PAGE) was titrated with 1 m*M* maltose in a VP-ITC calorimeter (MicroCal) at 25°C.

### Mutagenesis

Linker variants were generated by site-directed mutagenesis with a uracil template^38^ and transformed directly into *E. coli* BL21(DE3) cells (lacking pLysS) and plated on 24 cm × 24 cm LB-Agar plates with 60 μg/mL ampicillin. Mutagenic primers are listed in the Supporting Information.

### High-throughput screening

Individual colonies were picked with the aid of a QPix2^xt^ colony-picking robot (Genetix) into 96-well plates filled with 800-μL autoinduction media and 100 μg/mL ampicillin and grown with rapid (900 RPM) shaking overnight at 30°C. One hundred microliters of cell culture was removed and archived. The remaining cells were harvested by centrifugation (3000*g*) and frozen. Protein was extracted by freeze-thaw lysis and rapid shaking in 800-μL phosphate-buffered saline. Crude lysate was clarified by centrifugation at 4000*g* for 30 min. One hundred microliters of clarified lysate was transferred to flat-bottom black 96-well plates (Greiner) for fluorescence measurement. Fluorescence was assayed in a Safire^2^ plate-reading fluorimeter with stacker (Tecan). Excitation/emission wavelengths (nm) were as follows: blue (383/448), cyan (433/475), green (485/515), and yellow (515/527). Gain was 100 V and bandpasses were 5 nm. Fluorescence was measured again after addition of 10-μL 0.1*M* maltose (final maltose concentration 10 m*M*). After calculation of Δ*F*/*F* (*F*_maltose_ − *F*_o_)/*F*_o_, interesting candidates were streaked on agar plates from archived aliquots, grown for DNA preparation, and sequenced.

### Imaging

*E. coli* BL21(DE3) bacteria were transformed with a plasmid encoding either EGFP, PPYF, or PPYF.T203V, grown overnight in liquid autoinduction media at 30°C, harvested by centrifugation at 500*g*, and resuspended in Tris-buffered saline (TBS). They were diluted 1:10 in TBS and immobilized on standard microscope slides precoated with poly-l-lysine (Sigma) by centrifugation at 100*g* for 10 min. Excess bacteria were rinsed off with TBS. A coverslip was placed over the microscope slide and observed on an AxioImager fluorescence microscope (Zeiss) with a 60×/0.8 NA plan apochromatic objective lens (Zeiss). Exposure time was 1 s for EGFP and 6 s for PPYF and PPYF.T203V. Bacteria were reimaged after application of 10 m*M* maltose solution to the junction of the slide and coverslip; capillary action and diffusion were assumed to bring the final maltose concentration to a level that saturated the sensor. Fluorescence intensity was quantified by defining a region of interest around individual bacteria and determining the highest gray-value pixel intensity.

HEK293 cells were transiently transfected with a plasmid encoding the MBP-cpGFP165.PPYF.T203V gene fused to the C-terminus of PDGF receptor (a modified version of pDisplay lacking the hemaglutinin epitope tag). After 2 days of incubation at 37°C in 35-mm microwell dishes on No.1.5 coverslips, cells were gently rinsed with Hanks' Balanced Salt Solution (Gibco) and imaged with a modified Olympus BX microscope controlled with ScanImage software18 (http://www.ephus.org) and an Olympus 60×/0.9 NA LUMPlanFl/IR objective. Two-photon fluorescence excitation was evoked with a laser (Chameleon Vision II; Coherent, Santa Clara, CA) tuned to 910 nm. Fluorescence emission was passed through a 565-nm dichroic mirror and BG22 emission filter and measured using a photomultiplier tube (Hamamatsu, Hamamatsu City, Japan). Images were acquired at a resolution of 512 × 512 pixels at two frames per second. For maltose titration experiments, images were obtained at 10-s intervals for a duration of 2 min at each concentration. The steady-state fluorescence level at each concentration was calculated from images acquired in the second minute only.

### Two-photon spectroscopy

A two-photon excitation spectrum was obtained for 5 μ*M* solutions of each of the spectral variants, in the absence or presence of 0.1 m*M* maltose, excited by pulses from a mode-locked Ti:Sapphire laser (Chameleon Ultra, Coherent; operating at 80 MHz with a nominal 140-fs pulse width). An average laser power of 1 mW was delivered to the samples at the focus of a 60×/1.2 NA Water objective (UplanSApo, Olympus), and the fluorescence emission of the proteins was detected by an avalanche photodiode (PDM Series, Micro Photon Devices). The emission filters used were as follows: MBP165-cpAzurite and MBP165-cpCFP: 470 nm with 40-nm bandpass (ET470/40, Chroma Technology); MBP165-cpGFP and MBP165-cpYFP: 550 nm with 88-nm bandpass (Brightline fluorescence filter 550/88, Semrock). For a detailed presentation of the spectroscopic method, see “Excitation spectra and brightness optimization of two-photon excited probes,” Mutze *et al*., submitted to *Biophysical Journal*.

### X-ray crystallography

Maltose sensor proteins were expressed and purified as described above with an additional size exclusion chromatography purification step using a Superdex 200 column (GE Healthcare). Sensor proteins were crystallized using the vapor diffusion method. Details of crystallization conditions, data collection, and model refinement are given in the Supporting Information. The final models are of high quality with good stereochemistry (Supporting Information Table SI) and include most amino acids except the affinity tags, a few residues at the N- and C-termini, and the glycine-containing linkers connecting the two halves of the cpGFP domains.

## RESULTS

### Designing the MBP-cpGFP sensors: identification of cpGFP insertion sites in MBP

We hypothesized that allosteric coupling of ligand binding to fluorescence would require (a) that the site in MBP into which cpGFP is inserted must have the capacity to transduce the global conformational change of MBP to the local environment of the chromophore in cpGFP and (b) that the local environment of the chromophore must be optimized to increase the difference between the fluorescence of apo and the maltose-bound states of the protein.

**Figure 1:**
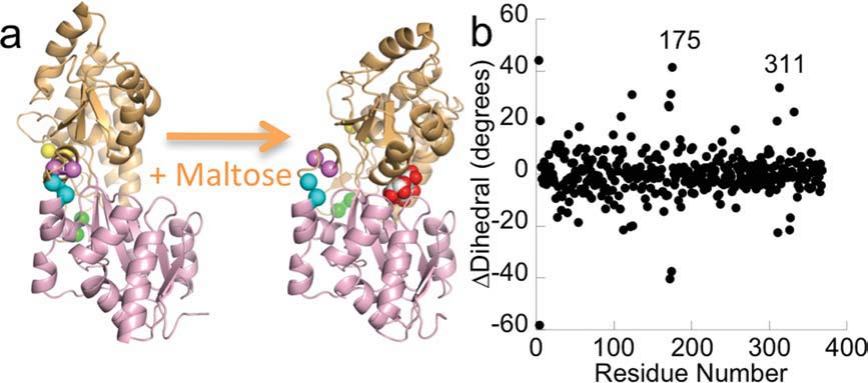
Conformational changes in MBP upon maltose binding. Cartoon representation (**a**). The residues between which cpGFP was inserted are identified with colored spheres at the Cα positions. Yellow: 165–166, green: 175–176, cyan: 311–312, and violet: 317–318. Figure was made with PyMOL.^41^ Analysis of backbone structural changes (**b**). The Cα dihedral is calculated from the four atoms: Cα_*i*+2_, Cα_*i*+1_, Cα_*i*_, Cα_*i*−1_. ΔDihedral is calculated as the difference in dihedrals between the closed (1ANF) and open (1OMP) states of MBP and corrected to fall within a range of −180° to 180°. The regions near residues 175 and 311 are labeled. There is a crystallographic artifact at the N-terminus resulting in the appearance of significant structural changes.

To identify viable insertion sites, we used the crystal structures of the maltose-bound, closed form of MBP^39^ (PDB 1ANF) and the ligand-free, open form of MBP^40^ (PDB 1OMP) [Fig. 1(a)] to guide rational design of MBP-cpGFP fusions that would result in maltose-dependent GFP fluorescence. Typical metrics for quantifying local structural perturbation, such as changes in solvent-accessible surface area or distance difference matrices, identify residues for which the change in local environment is defined by the three-dimensional relationship between residues, but not their sequential relationship. Thus, we analyzed the change in dihedral angle (defined by the Cα atoms spanning four residues) to identify maltose-dependent structural changes in sequentially adjacent residues [Fig. 1(b)]; this analysis shows that the Cα chain is “torqued” around residues 175 (ΔDihedral = +41°) and 311 (ΔDihedral = −22°) upon ligand binding. We hypothesized that this sequential conformational change could be coupled to structural changes of an inserted cpGFP, resulting in “GCaMP-like”^27^ maltose-dependent fluorescence for the fusion protein.

In a previous study, the genes for circularly permuted β-lactamase (cpBla) and MBP were randomly digested and reassembled to create a library of MBP-cpBla chimeras.^42^, ^43^ MBP-cpBla fusions that resulted in maltose-dependent β-lactamase activity were selected by growth on ampicillin, and proteins with insertions of cpBla at MBP residues 165 and 317 were identified.^42^, ^43^ As the ΔDihedral of MBP165 is +11° (moderate change) and MBP317 is +2° (no real change), we constructed all four MBP-cpGFP templates to test our predictive method and the interchangeability of cpBla and cpGFP at sites identified from the MBP-cpBla screen. We would not expect a simple metric like ΔDihedral to capture all the intricacies of allosteric coupling but rather would reduce the number of potential frameworks used for linker optimization to an experimentally tractable number. For the fluorescent reporter, we used the circularly permuted variant of GFP present in GCaMP, cpGFP146^44^ (here on called cpGFP). We used PCR assembly to construct fusion proteins with -GlyGly- linkers between MBP and each terminus of cpGFP. (For detailed amino acid sequence information, see Supporting Information Figs. S1–S4.)

### Designing the MBP-cpGFP sensors: optimizing linkers for MBP165-, 175-, 311-, and 317-cpGFP

After initial testing of these four sensors, we generated libraries of variants with randomized linkers (Supporting Information Fig. S5) by single-stranded uracil template mutagenesis^38^ and screened several thousand variants in semi-high-throughput fashion, measuring fluorescence intensity of clarified cell lysate in the absence and presence of 10 m*M* maltose.

A fusion protein in which cpBla replaces residue 317 of MBP (MBP317-cpBla) has strong maltose-dependent “switching”^42^, ^43^, ^45^, ^46^; thus, one might expect insertion of cpGFP at residue 317 to result in maltose-dependent fluorescence. However, a screen of ∼1000 linker variants yielded no fluorescent sensor proteins (Supporting Information Fig. S6) even though the framework protein still bound maltose, as determined by isothermal titration calorimetry (Supporting Information Fig. S7).

The other fusion protein that was fruitful for the creation of maltose-dependent β-lactamase switches was an insertion of cpBla at residue 165 of MBP.^43^ Insertion of cpGFP at residue 165 of MBP (MBP165-cpGFP) with -GlyGly- linkers flanking the cpGFP resulted in a protein in which fluorescence increased 20% (Δ*F*/*F* = 0.2) upon addition of saturating maltose. Screening a fully degenerate, length-two library (XX) at either the MBP-cpGFP linker (linker 1) or the cpGFP-MBP linker (linker 2) yielded proteins with maltose-dependent fluorescent increases > 300% or decreases > 50% (Supporting Information Fig. S6). Many of the variants with increased Δ*F*/*F* values had linkers containing proline(s). Subsequent libraries constructed from oligonucleotides encoding XP or PX and randomization of the residues in GFP from residues 146 to 150 were screened, yielding a final variant with: a two-proline MBP-cpGFP linker, a two-glycine cpGFP-MBP linker, GFP-H148Y, and GFP-Y151F. This variant, called “MBP165-cpGFP.PPYF” (abbreviated PPYF), has a Δ*F*/*F* = 2.5 and a *K*_d_ for maltose of 3 μ*M*.

Our structural analysis shows that the Cα chain is “torqued” around residues 175 and 311 upon ligand binding [Fig. 1(b)]; thus, we created fusion proteins with cpGFP inserted at these positions and screened the linkers as before. Approximately 400 “XX” variants of linkers 1 and 2 for the MBP311-cpGFP protein were screened (Supporting Information Fig. S6). This yielded variant MBP311-cpGFP.L2-NP (-AsnPro- at linker 2), which has a Δ*F*/*F* of 1.0 and a *K*_d_ for maltose of 2 μ*M*. This variant has an inferior maltose-dependent fluorescence increase than PPYF, but demonstrates generality of the cpFP insertion method.

MBP175-cpGFP was also screened with XX linkers, and a few variants with Δ*F*/*F* ≍ 1 were identified (Supporting Information Fig. S6). One mutant, with the first linker encoding HL (MBP175-cpGFP.L1-HL), has a Δ*F*/*F* = 0.5 and a *K*_d_ for maltose of 1.3 μ*M*.

We cannot predict *a priori* how the residues in the proximity of the chromophore in cpGFP will affect fluorescence of the apo and maltose-bound states, and thus we cannot predict whether a given linker composition will result in sensors with high or low Δ*F*/*F*. However, our observation that the three insertion positions with absolute ΔDihedral values greater than 10° (165, 175, and 311) could be made into good sensors by linker optimization combined with the observation that the one position with a ΔDihedral of only 2° (317) failed to be made into a good sensor leads us to believe that choice of insertion site by structural analysis is preferable to random insertion.

### Changing the ligand-binding and fluorescent properties of the sensors: ligand-binding affinity

One objective in the development of generic biosensors is for the framework to permit independent optimization of binding and signaling properties. We systematically tested this notion in the high-SNR sensor PPYF, by rationally altering maltose-binding affinity, changing the ligand-binding specificity from maltose to sucrose, and creating a family of sensors in multiple colors.

As a first step, the effects of mutation of three tryptophan side chains in the maltose-binding pocket (W230, W62, and W340) were tested. These have previously been shown to lower the affinity of MBP for maltose by one, two, or three orders of magnitude, respectively, when mutated to alanine.^47^ A mutation to the hinge region, I329W, was also made to PPYF, as this has been shown to increase maltose affinity by about twofold in both wild-type MBP^48^ and in the MBP-cpBla switches.^42^, ^45^ For the PPYF sensor, the three tryptophan-to-alanine binding-pocket mutations behave as expected, lowering affinity by between one and three orders of magnitude (Supporting Information Fig. S8). However, the I329W mutation does not increase affinity as expected, but rather decreases it, and also decreases Δ*F*/*F* (Supporting Information Fig. S8). We take this unexpected result to suggest that the mechanism of fluorescence change in this sensor is dependent on subtle interactions between MBP and cpGFP that are linked to the I329W mutation.

### Changing the ligand-binding and fluorescent properties of the sensors: ligand-binding specificity

Previously, it has been reported that mutation of four residues in the binding pocket of MBP (A63H, R66H, Y155E, and W340E) could turn a maltose-sensing protein (with an exogenous cysteine-attached fluorophore) into a zinc-sensing protein.^49^ The mutations of that zinc-binding design (A63H, R66H, H155E, and W340E; the protein called “A*” in Ref.^49^) were made to the PPYF scaffold. There was no observable change in fluorescence upon addition of Zn^2+^ or EDTA to that protein (data not shown). It is not clear why no fluorescence response was seen, and we did not investigate this construct further.

As an alternative test for changing the ligand-binding specificity of the sensor while preserving fluorescence signaling, the “5-7” mutations (D14L, K15F, W62Y, and E111Y), previously shown to confer MBP with an affinity for sucrose,^35^ were made to PPYF. In the previous study, these four mutations conferred wild-type MBP and MBP317-cpBla with 6 and 1 μ*M* affinity for sucrose (respectively) and both with ∼20 μ*M* affinity for maltose (wild-type and cpBla-MBP have 1.0 and 0.5 μ*M* affinity for maltose, and no detectable affinity for sucrose). Here, the 5-7 mutations in the context of PPYF instead conferred ∼2 m*M* affinity for sucrose and ∼3 m*M* affinity for maltose [Supporting Information Fig. S9(a)]. To address the apparent discrepancy between expected (micromolar) and observed (millimolar) affinity for disaccharides, the 5-7 mutations were made to sensors with cpGFP inserted at different positions in MBP and with different linker compositions. In the context of MBP165-cpGFP.PCF, the 5-7 mutations confer very low (but observable) binding preference for sucrose over maltose [Supporting Information Fig. S9(b)]. The trend of higher (but still weak) affinity for sucrose (∼0.6 m*M*) over maltose (∼6 m*M*) continues when the 5-7 mutations are made in the context of MBP175-cpGFP.L1-HL [Supporting Information Fig. S9(c)]. In the context of MBP311-cpGFP.L2-NP, the 5-7 mutations appear to eliminate all binding [Supporting Information Fig. S9(d)]. The preference for sucrose over maltose of the 5-7 variants of the sensors is consistent with the binding properties of the 5-7 variants of MBP alone and MBP-cpBla.^35^ The lower affinity for both ligands of the 5-7 variants of the sensors may be the consequence of the inserted cpGFP shifting the open and closed equilibrium.

### Changing the ligand-binding and fluorescent properties of the sensors: sensor color

**Figure 2:**
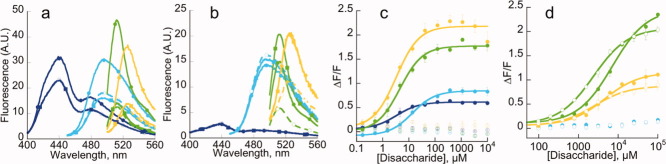
Fluorescence emission spectra of the MBP165-blue, cyan, green, and yellow wild-type sensors (**a**) and the 5-7 variants (**b**) in the absence of ligand (dashed lines, open circles), with 10 m*M* maltose (solid lines, filled circles) or 10 m*M* sucrose (solid lines, filed squares). Sensors were excited at 383, 433, 485, and 485 nm, respectively. Titration of maltose and sucrose in the blue, cyan, green, and yellow MBP165 wild-type sensors (**c**) and for the 5-7 variants (**d**). Filled circles are titration of maltose, and open circles are titration of sucrose. For the wild-type sensors, *K*_d_s for maltose binding are as follows: blue 3.3 μ*M*, cyan 13 μ*M*, green 4.5 μ*M*, and yellow 3.3 μ*M*. No sucrose binding is observed. For the 5-7 variants, *K*_d_ of green is 2.4 m*M* (sucrose) and 7.1 m*M* (maltose). *K*_d_ of yellow is 2.5 m*M* (sucrose) and 4.5 m*M* (maltose). The fluorescence and signal-to-noise ratio of the cyan and blue 5-7 variants are so low that it is not possible to accurately determine Δ*F*/*F*.

The color of GFP can be altered by changing the amino acids that either comprise or interact with the chromophore (reviewed in Ref.^50^). Using PPYF as a template, Y66W (to yield a cyan variant^51^; “cpCFP”), L64F+T65G+V68L+T203Y (yellow^51^; “cpYFP”), and Y66H (blue^51^; “cpBFP”) mutations were made. The new proteins have fluorescence emission spectra consistent with their respective intended designs [Fig. 2(a)]. The Δ*F*/*F* of these proteins in response to maltose is different (in each case inferior) from the Δ*F*/*F* of 2.5 observed in PPYF-green. The chromatic variants have different molecular structures, and there is no reason to expect that the “PPYF” sequence is ideal for any chromophore other than the cpGFP for which it was screened. The MBP165-cpYFP.PPYF sensor, which has the same covalent chromophore structure as PPYF, has the greatest Δ*F*/*F* of the three spectral variants [Fig. 2(a)]. MBP165-cpCFP.PPYF has a lower Δ*F*/*F* than the green and yellow variants, but by incorporating previously identified mutations (L1-PC + GFP-Y151F; the resulting protein is called MBP165.cpCFP.PCF), a variant with Δ*F*/*F* = 0.8 was obtained [Fig. 2(a)]. The MBP165-cpBFP.PPYF variant, while dimly fluorescent, is not a sensor, and a screen of 800 linker variants failed to produce any variant with Δ*F*/*F* > 0.2 (Supporting Information Fig. S10). As MBP165-cpBFP.PPYF was very dim, the Azurite mutations T65S+V150I+V224R^52^ were included, in the hope that they would increase brightness and stability, and make MBP165-cpAzurite a good template for linker screening. Using oligonucleotides encoding XX amino acid linkers, a variant was obtained, MBP165-cpAzurite.L2-FE, that had Δ*F*/*F* = 0.8 [Fig. 2(a)].

### Changing the ligand-binding and fluorescent properties of the sensors: simultaneous alteration of sensor color and ligand specificity/affinity

To complete the demonstration of modular manipulation of sensor color and ligand binding, the four sucrose-binding “5-7” mutations described above that conferred weak sucrose affinity in the green sensor (MBP165-cpGFP.PPYF) were made to the blue, cyan, and yellow maltose sensors (MBP165-cpAzurite.L2-FE, MBP165-cpCFP.PCF, and MBP165-cpYFP.PPYF). The green and yellow sensors showed increased fluorescence upon addition of 10 m*M* sucrose, but the cyan and blue proteins did not [Fig. 2(b)]. Like the green variant, the yellow variant has no detectable sucrose affinity with the wild-type binding pocket [Fig. 2(c)] and millimolar affinity for both sugars, with preference for sucrose over maltose [Fig. 2(d)]. The low affinity of these sensors will most likely make them best suited for applications in which sucrose concentrations are expected to be in the millimolar range, such as monitoring the flux of sugars in tubers.^53^ Although further efforts to redesign MBP for sucrose-binding specificity might yield a higher affinity sensor, in practice, creating an intensity-based sucrose sensor would probably be more easily accomplished by inserting cpGFP into a sugar-binding protein with more appropriate sucrose-binding properties.^54^

**Figure 3:**
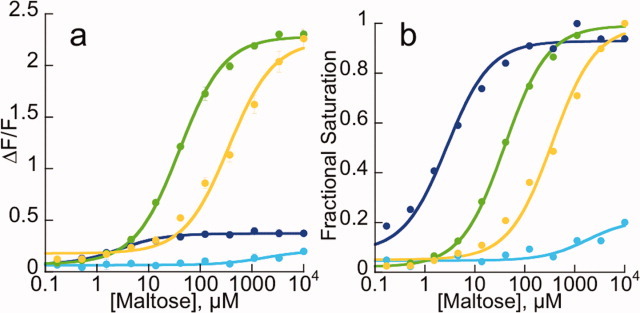
Four-colored composite maltose sensor. The four-colored maltose sensors, with appropriate mutations to the maltose-binding site, were combined in one solution to detect changes in maltose concentration from less than 1 μ*M* to greater than 1 m*M*. Blue (wt-binding pocket) has an affinity of 2.7 μ*M*. Green (W230A) has an affinity of 40 μ*M*. Yellow (W62A) has an affinity of 350 μ*M*. Cyan (W340A) has an affinity of ∼1.7 m*M*. Data are plotted at Δ*F*/*F* (**a**) or normalized to fractional saturation (**b**).

On the other hand, the tryptophan-to-alanine mutations described above can be used to create a family of sensors that can determine maltose concentrations from 1 μ*M* to over 1 m*M* by mixing four colored variants with different affinities for maltose. Using the native binding-site residues in the blue maltose sensor (MBP165-cpAzurite-L2-FE), the W230A mutation in the green maltose sensor (MBP165-cpGFP.PPYF.W230A), the W62A mutation in the yellow maltose sensor (MBP165-cpYFP.PPYF.W62A), and the W340A mutation in the cyan maltose sensor (MBP165-cpCFP.PCF.W340A), and mixing the four proteins together in one solution, the blue, cyan, green, and yellow fluorescence channels can be simultaneously monitored to determine maltose concentration. As seen in Figure 3, as maltose concentration increases, the blue sensor increases in fluorescence first (*K*_d_ ∼2.7 μ*M*), then the green (*K*_d_ ∼40 μ*M*), then the yellow (*K*_d_ ∼350 μ*M*), and at high maltose concentrations, the cyan variant begins to increase its fluorescence (*K*_d_ ∼1.7 m*M*). If the blue and cyan sensors were further optimized for greater changes in fluorescence, the high dynamic range composite sensor would be even more robust.

### Potential *in vivo* application of intensity-based sensors: imaging bacteria

The ultimate value of genetically encoded fluorescent sensors is in their utility for observing analyte flux in living cells and organisms. In a simple proof-of-principle experiment, *E. coli* bacteria expressing PPYF or PPYF.T203V (see “second-generation maltose sensors” below) were imaged in the green fluorescence channel in the absence of maltose and then reimaged after addition of saturating maltose to the media. The bacteria expressing the sensors clearly became brighter, whereas control bacteria expressing EGFP appeared unchanged (Supporting Information Fig. S11). This increase in fluorescence was quantified by measuring the peak (gray-value) pixel intensity of each bacterium. Those expressing PPYF undergo an approximate doubling of fluorescence (bacterium-averaged Δ*F*/*F* = 1.1 ± 0.4), those expressing PPYF.T203V have slightly increased Δ*F*/*F* (Δ*F*/*F* = 1.29 ± 0.2), whereas those expressing EGFP have no change in fluorescence (Δ*F*/*F* = −0.01 ± 0.05) (Supporting Information Table SI).

### Potential *in vivo* application of intensity-based sensors: two-photon imaging of mammalian cells

Multiphoton microscopy^55^ has opened new frontiers for *in vivo* fluorescence imaging,^56^ in particular neuronal activity visualization through the use of genetically encoded calcium indicators.^19^ To demonstrate that the maltose sensors described here have the potential to be used for two-photon imaging, we collected fluorescence excitation spectra (Supporting Information Fig. S12). With a 535-nm bandpass emission filter (50-nm bp), MBP165-cpGFP.PPYF showed a 10-fold maltose-dependent increase in fluorescence when excited at 940 nm. All four spectral variants showed a significant maltose-dependent increase in two-photon fluorescence, but in some cases the use of off-peak emission bandpass filters dramatically reduced the magnitude (see Methods).

**Figure 4:**
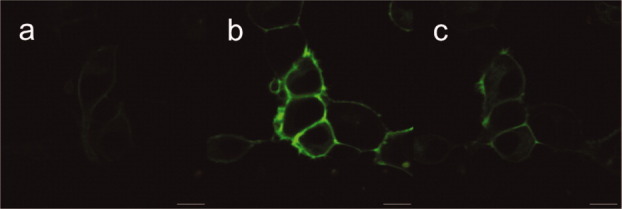
Images of individual HEK293 cells expressing membrane-displayed PPYF.T203V in the absence of maltose (**a**), in the presence of 1 m*M* maltose (**b**), and after washout with maltose-free buffer (**c**). Scale bars are 10 μm.

In another proof-of-principle experiment, we cloned MBP165-cpGFP.PPYF.T203V (see “second-generation maltose sensors” below) into a modified version of the pDisplay vector (Invitrogen) for extracellular display on the surface of transiently transfected human embryonic kidney (HEK293) cells. The sensor localizes to the plasma membrane (Fig. 4) and increases in brightness in a concentration-dependent manner when perfused with buffers of varying maltose concentration. The Δ*F*/*F* is 5.8-fold, very close to that of the soluble protein produced in *E. coli* (see below), with the midpoint of the maltose-dependent fluorescence increase being 6.5 μ*M* [Supporting Information Fig. S13(a)], very similar to the affinity determined on purified protein (5 μ*M*). Furthermore, the surface-displayed sensor responds rapidly to a pulse of 1 m*M* maltose [Supporting Information Fig. S13(b)], indicating that the time course for its action is useful for transient events.

### Crystal structure analysis of maltose sensors

**Figure 5:**
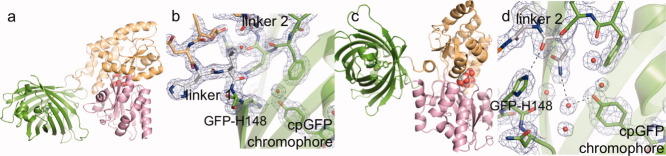
Cartoon representation and close-up views of interdomain linkers and selected amino acids of the cpGFP chromophore environment of the structure of MBP175-cpGFP.L1-HL (**a**, **b**) and MBP311-cpGFP.L2-NP (**c**, **d**) bound to maltose. The MBP domain is colored as in Figure 1(a). The cpGFP domain is green, and the interdomain linkers are colored white. The cpGFP chromophore is displayed as sticks and the bound maltose as red and white spheres. Ordered water molecules are represented as red spheres. Selected hydrogen bonds are displayed as dashed black lines. β-Strands 10 and 11 of cpGFP are displayed as semitransparent for clarity. The 2*F*_o_ − *F*_c_ electron density map calculated with the displayed residues omitted from the model is shown as blue mesh.

To learn about the conformations of the interdomain linkers and the molecular mechanism of sensing, we determined high-resolution structures of several of the maltose sensors created here. We set up crystallization trials with MBP165-cpGFP.PPYF, MBP175-cpGFP.L1-HL, and MBP311-cpGFP.L2-NP in the presence and absence of excess maltose, from which both MBP175-cpGFP.L1-HL and MBP311-cpGFP.L2-NP crystallized in the presence of maltose. We solved the X-ray structures of these sensors to 1.9 and 2.0 Å resolution, respectively, by molecular replacement [Fig. 5(a,c)]. As expected, the structures of the cpGFP and MBP domains of the sensors are superimposable with published crystal structures of cpGFP (from GCaMP2; RMSD = 0.36 and 0.38 Å, respectively, for comparing 221 common Cα atoms) and MBP-maltose (RMSD = 0.43 and 0.37 Å, 370 Cα). The structure of MBP is largely unperturbed by insertion of the cpGFP domain; only residues around the 175 and 311 insertion sites show any significant displacement. GFP-H148, which H-bonds the GFP chromophore in the structure of native GFP, also directly H-bonds the chromophore in the MBP175-cpGFP.L1-HL-maltose structure [Fig. 5(b)], although a different rotamer is observed. In the MBP311-cpGFP.L2-NP-maltose structure, GFP-H148 is pulled away from the chromophore and is largely replaced by the Asn from linker 2, which makes H-bond interactions to both strand 8 of the GFP barrel and the chromophore phenolate oxygen [through a water molecule, Fig. 5(d)]. GFP-H148, meanwhile, seems to stabilize the conformation of linker 2 of MBP311-cpGFP.L2-NP by H-bonding the backbone carbonyl of the linker 2 Asn. There is some solvent access to the cpGFP chromophore through the hole in the GFP barrel created by circular permutation, although the interdomain linkers block much of the opening in both structures. Relatively few contacts are made between the cpGFP and MBP domains.

Based on the structures of two maltose-bound sensors, the sensing mechanism likely involves a shift in the relative position of linker 1 and linker 2 induced by the conformational change in the MBP domain associated with maltose binding [Fig. 1(a)]. The register shift of interactions between the two linkers could alter the proximity of linker 2 and nearby side chains to the cpGFP chromophore and change the water structure in the cpGFP opening, leading to a shift in the chromophore protonation equilibrium. This might explain why rigid proline is preferred in either linker, as conformational changes upon ligand binding might be better propagated through the rigid linkers to the cpGFP chromophore environment.

### Epilog: second-generation maltose sensors

The primary strategy we used to increase Δ*F*/*F* for the maltose sensors was to alter the local environment of the chromophore of cpGFP by randomizing the MBP-cpGFP and cpGFP-MBP linkers and screening for improved variants. In a separate effort to make improvements to the brightness and Δ*F*/*F* of GCaMP, the local environment of the chromophore was altered by randomizing residues within cpGFP and screening for improved variants.^19^ One of these mutants, GFP-T203V, decreases the fluorescence emission of the apo state and increases that of the saturated state in GCaMP2 (see Fig. 1(c) in Ref.^19^). In the context of MBP165-cpGFP.PPYF, the T203V mutation decreases the fluorescence emission of the apo state by half [Supporting Information Fig. S14(a)], whereas saturated fluorescence and affinity are unchanged [Supporting Information Fig. S14(b)], increasing Δ*F*/*F* to 6.5. In the maltose-saturated state, PPYF itself has about a quarter the brightness of EGFP and half the brightness of cpGFP [Supporting Information Fig. S14(a)].

However, in the context of MBP311-cpGFP.L2-NP, the T203V mutation decreases the brightness of both the apo state and the saturated-state equally, resulting in no significant change in Δ*F*/*F* [Supporting Information Fig. S14(c,d)]. This result indicates that the benefits of the T203V mutation are not universally transferable, and that cpGFP-based fluorescent sensors need to be optimized individually.

## SUMMARY AND CONCLUSIONS

We demonstrate the systematic construction of a family of intensity-based biosensors from circularly permuted FPs and a PBP. Our strategy entailed analysis of the structure of the binding protein to identify points in the amino acid sequence into which a cpFP can be inserted in such a way that the ligand-induced conformational change of the PBP results in a change in the local environment of the FP chromophore. The ideal composition of the residues that comprise the environment of the chromophore is not obvious, and thus libraries of variants at residues near the chromophore (the PBP-cpFP and cpFP-PBP linkers as well as nearby positions) are screened to identify those variants that have significant changes in fluorescence emission intensity upon binding. We have demonstrated that both the ligand-binding properties and the sensor color can be changed, largely independently, by mutation of the residues comprising the binding site or the chromophore, respectively.

We demonstrated the *in vivo* utility of the engineered sensors to visualize maltose transport across the plasma membrane of individual *E. coli* bacteria and to detect extracellular maltose in mammalian cell cultures. Further experiments are necessary to determine whether the sensors will work similarly well in living animals at body temperature. All four colors of engineered sensors were sufficiently bright under both one-photon and two-photon excitation, with adequate Δ*F*/*F* and SNR, to permit preliminary experiments in intact animals. Intriguingly, the performance of some sensors was significantly better under multiphoton excitation.

The bacterial PBP superfamily contains proteins specific for glutamate, glutamine, glycine, other amino acids, GABA, acetylcholine, diverse carbohydrates, peptides, lactate, and Zn^2+^, among numerous other analytes.^30^ Further diversification of the ligand-binding properties of the family by rational design^57^ or directed evolution^58^ may provide additional interesting specificities (e.g., histamine from histidine-binding protein). Sensors for each of these target molecules would be of great utility in visualizing neurotransmission, cellular metabolism, and disease states in cells and intact organisms. We expect the methods outlined here to be useful for the development of sensors from other members of the PBP family and to other classes of allosteric proteins as well. In a separate report (Guevara *et al*., manuscript in preparation), we have applied these methods to create a sensor from the phosphonate-binding protein (PhnD) of *E. coli*. Furthermore, ongoing diversification of the fluorescent-protein family may provide for the creation of red-shifted sensors, facilitating deep imaging in biological tissues.

We found it difficult to predict *a priori* the precise FP insertion sites, linker sequences, and surrounding protein mutations that would give rise to optimal sensors. However, we were able to rank-order potential insertion sites based on a simple geometric estimate and found that highly ranked positions gave rise to high-SNR sensors after optimization. A combination of rational design and random screening produced the best results—the precise effect of mutations on fluorescence readout is difficult to predict, as fluorescence is particularly sensitive to small perturbations.^59^ Mutations to the binding and FPs were found to be largely independent (i.e., chromophore changes induced the expected colors; grafting literature mutations on the binding pocket conveyed the anticipated binding specificity). However, the precise nature of the binding and fluorescence properties produced a number of unanticipated results (i.e., allosteric hinge mutations did not graft easily; binding pocket mutations produced sensors with lower-than-expected affinity), illustrating that each sensor will likely have to be specifically optimized by rational design and directed evolution.

## Abbreviations

CaM: calmodulin
cpBla: circularly permuted β-lactamase
cpGFP: circularly permuted GFP
FP: fluorescent protein
FRET: fluorescence resonance energy transfer
GFP: *Aequorea victoria* green fluorescent protein
MBP: *E. coli* maltose-binding protein
PBP: periplasmic binding protein
SNR: signal-to-noise ratio
