# Supplementary material for: A genetically encoded, high-signal-to-noise maltose sensor
Source: Proteins. 2011 Oct 12;79(11):3025–36. doi: 10.1002/prot.23118 (PMC3265398; doi:10.1002/prot.23118)
Supplement: Supplementary file 1 [file prot0079-3025-SD1.pdf]

Fig. S1. MBP165-cpGFP amino acid sequence

MBP165-cpGFP

```
pRSET Leader | MBP 1-165
MRGSHHHHHHGMASMTGGQQMGRDLYDDDDKDRWGSKIEEGKLVIWINGDKGYNGLAEVGKKFEKDTGIKVTVEHPDKLE 80

MBP 1-165
EKFPQVAATGDGPDIIFWAHDRFGGYAQSGLLAEITPDKAFQDKLYPFTWDAVRYNGKLIAYPIAVEALSLIYNKDLLPN 160

MBP 1-165 | cpGFP 147-238
PPKTWEEIPALDKELKAKGKSALMFNLQEPYFTWPLIAADGGSHNVYIMADKQKNGIKANFKIRHNIEDGGVQLAYHYQQ 240

cpGFP 147-238 | Linker | cpGFP 1-146
NTPIGDGPVLLPDNHYLSTQSKLSKDPNEKRDHMVLLEFVTAAGITLGMDELYKGGTGGSMVSKGEELFTGVVPILVELD 320

cpGFP 1-146
GDVNGHKFSVSGEGEGDATYGKLTLKFICTTGKLPVPWPTLVTTLTYGVQCFSRYPDHMKQHDFFKSAMPEGYIQERTIF 400

cpGFP 1-146 | MBP 166-370
FKDDGNYKTRAEVKFEGDTLVNRIELKGIDFKEDGNILGHKLEYNFNGGYAFKYENGKYDIKDVGVDNAGAKAGLTFLVD 480

MBP 166-370
LIKNKHMNADTDYSIAEAAFNKGETAMTINGPWAWSNIDTSKVNYGVTVLPTFKGQPSKPFVGVLSAGINAASPNKELAK 560

MBP 166-370
EFLENYLLTDEGLEAVNKDKPLGAVALKSYEEELVKDPRIAATMENAQKGEIMPNIPQMSAFWYAVRTAVINAASGRQTV 640

MBP 166-370
DEDLKDAQTRITKGSHHHHHHG.
```

MBP165-cpGFP.PPYF

```
MBP 1-165 | cpGFP 147-238
PPKTWEEIPALDKELKAKGKSALMFNLQEPYFTWPLIAADPPSYNVFIMADKQKNGIKANFKIRHNIEDGGVQLAYHYQQ
```

MBP165-cpGFP.PCF

```
MBP 1-165 | cpGFP 147-238
PPKTWEEIPALDKELKAKGKSALMFNLQEPYFTWPLIAADPCSHNVFIMADKQKNGIKANFKIRHNIEDGGVQLAYHYQQ
```

Fig. S2. MBP175-cpGFP amino acid sequence

MBP175-cpGFP

pRSET Leader | MBP 1-175

MRGSHHHHHHGMASMTGGQQMGRDLYDDDDKDRWGSKIEEGKLVIWINGDKGYNGLAEVGKKFEKDTGIKVTVEHPDKLE 80

MBP 1-175

EKFPQVAATGDGPDIIFWAHDRFGGYAQSGLLAEITPDKAFQDKLYPFTWDAVRYNGKLIAYPIAVEALSLIYNKDLLPN 160

MBP 1-175 | cpGFP 147-238

PPKTWEEIPALDKELKAKGKSALMFNLQEPYFTWPLIAADGGYAFKYENGGSHNVYIMADKQKNGIKANFKIRHNIEDGG 240

cpGFP 147-238 | Linker | cpGFP 1-146

VQLAYHYQQNTPIGDGPVLLPDNHYLSTQSKLSKDPNEKRDHMVLLEFVTAAGITLGMDELYKGGTGGSMVSKGEELFTG 320

cpGFP 1-146

VVPILVELDGDVNGHKFSVSGEGEGDATYGKLTLKFICTTGKLPVPWPTLVTTLTYGVQCFSRYPDHMKQHDFFKSAMPE 400

cpGFP 1-146 | MBP 176-370

GYIQERTIFFKDDGNYKTRAEVKFEGDTLVNRIELKGIDFKEDGNILGHKLEYNFNGGKYDIKDVGVDNAGAKAGLTFLV 480

MBP 176-370

DLIKNKHMNADTDYSIAEAAFNKGETAMTINGPWAWSNIDTSKVNYGVTVLPTFKGQPSKPFVGVLSAGINAASPNKELA 560

MBP 176-370

KEFLENYLLTDEGLEAVNKDKPLGAVALKSYEEELVKDPRIAATMENAQKGEIMPNIPQMSAFWYAVRTAVINAASGRQT 640

MBP 176-370

VDEDLKDAQTRITKGSHHHHHHG.

MBP175-cpGFP.L1-HL

MBP 1-175 | cpGFP 147-238

PPKTWEEIPALDKELKAKGKSALMFNLQEPYFTWPLIAADGGYAFKYENHLSHNVYIMADKQKNGIKANFKIRHNIEDGG

Fig. S3. MBP311-cpGFP amino acid sequence

MBP311-cpGFP

pRSET Leader | MBP 1-311

MRGSHHHHHHGMASMTGGQQMGRDLYDDDDKDRWGSKIEEGKLVIWINGDKGYNGLAEVGKKFEKDTGIKVTVEHPDKLE 80

MBP 1-311

EKFPQVAATGDGPDIIFWAHDRFGGYAQSGLLAEITPDKAFQDKLYPFTWDAVRYNGKLIAYPIAVEALSLIYNKDLLPN 160

MBP 1-311

PPKTWEEIPALDKELKAKGKSALMFNLQEPYFTWPLIAADGGYAFKYENGKYDIKDVGVDNAGAKAGLTFLVDLIKNKHM 240

MBP 1-311

NADTDYSIAEAAFNKGETAMTINGPWAWSNIDTSKVNYGVTVLPTFKGQPSKPFVGVLSAGINAASPNKELAKEFLENYL 320

MBP 1-311 | cpGFP 147-238

LTDEGLEAVNKDKPLGAVALKSYEEELGGSHNVYIMADKQRNGIKANFKIRHNIEDGGVQLAYHYQQNTPIGDGPVLLPD 400

cpGFP 147-238 | Linker | cpGFP 1-146

NHYLSTQSKLSKDPNEKRDHMVLLEFVTAAGITLGMDELYKGGTGGSMVSKGEELFTGVVPILVELDGDVNGHKFSVSGE 480

cpGFP 1-146

GEGDATYGKLTLKFICTTGKLPVPWPTLVTTLTYGVQCFSRYPDHMKQHDFFKSAMPEGYIQERTIFFKDDGNYKTRAEV 560

cpGFP 1-146 | MBP 312-370

KFEGDTLVNRIELKGIDFKEDGNILGHKLEYNFNGGAKDPRIAATMENAQKGEIMPNIPQMSAFWYAVRTAVINAASGRQ 640

MBP 312-370

TVDEDLKDAQTRITK.

MBP311-cpGFP.L2-NP

cpGFP 1-146 | MBP 312-370

KFEGDTLVNRIELKGIDFKEDGNILGHKLEYNFNNPAKDPRIAATMENAQKGEIMPNIPQMSAFWYAVRTAVINAASGRQ

Fig. S4. MBP317-cpGFP amino acid sequence

MBP317-cpGFP

pRSET Leader | MBP 1-317

MRGSHHHHHHGMASMTGGQQMGRDLYDDDDKDRWGSKIEEGKLVIWINGDKGYNGLAEVGKKFEKDTGIKVTVEHPDKLE 80

MBP 1-317

EKFPQVAATGDGPDIIFWAHDRFGGYAQSGLLAEITPDKAFQDKLYPFTWDAVRYNGKLIAYPIAVEALSLIYNKDLLPN 160

MBP 1-317

PPKTWEEIPALDKELKAKGKSALMFNLQEPYFTWPLIAADGGYAFKYENGKYDIKDVGVDNAGAKAGLTFLVDLIKNKHM 240

MBP 1-317

NADTDYSIAEAAFNKGETAMTINGPWAWSNIDTSKVNYGVTVLPTFKGQPSKPFVGVLSAGINAASPNKELAKEFLENYL 320

MBP 1-317 | cpGFP 147-238

LTDEGLEAVNKDKPLGAVALKSYEEELVKDPRSHNVYIMADKQKNGIKANFKIRHNIEDGGVQLAYHYQQNTPIGDGPVL 400

cpGFP 147-238 | Linker | cpGFP 1-146

LPDNHYLSTQSKLSKDPNEKRDHMVLLEFVTAAGITLGMDELYKGGTGGSMVSKGEELFTGVVPILVELDGDVNGHKFSV 480

cpGFP 1-146

SGEGEGDATYGKLTLKFICTTGKLPVPWPTLVTTLTYGVQCFSRYPDHMKQHDFFKSAMPEGYIQERTIFFKDDGNYKTR 560

cpGFP 1-146 | MBP 318-370

AEVKFEGDTLVNRIELKGIDFKEDGNILGHKLEYNFNAATMENAQKGEIMPNIPQMSAFWYAVRTAVINAASGRQTVDED 640

MBP 318-370

LKDAQTRITKGSHHHHHHG.

Fig. S5. Mutagenic primers used

165 Linker1 primers	165 Linker2 primers
PLIAADGxxNVYIM	KLEYNFNxxYAFKYEN
PLIAADxxNVYIM	KLEYNFNxYAFKYEN
PLIAADGGxxNVYIM	KLEYNFNYAFKYEN
PLIAADGxPNVYIMG	KLEYNFxYAFKYEN
PLIAADGIxNVYIMG	KLEYNxxYAFKYEN
PLIAADPxSHNVYIM	KLEYNWxYAFKYEN
PLIAADxPSHNVYIM	KLEYNxKYAFKYEN
PLIAADxxSHNVYIM	KLEYNFNPxYAFKYEN
PLIAADxxSHNVFIM	KLEYNFNxPYAFKYEN
PLIAADPxSHNVFIM	
PLIAADPxSYNVFIM	
PLIAADxxSYNVFIM	
PLIAADPxSYNVFIM	
PLIAADxxSYNVFIM	
PLIAADPxSxNVYIM	
PLIAADPxSHxVYIM	
PLIAADPxSHNxYIM	
PLIAADPxSHNVxIM	

175 Linker1 primers	175 Linker2 primers
AFKYENXXSHNVYIM	KLEYNFNXXKYDIKDV

311 Linker1 primers	311 Linker2 primers
KSYEELXXSHNVYIM	KLEYNFNXXAKDPRIA
KSYEELPXSHNVYIM	KLEYNFNPXAKDPRIA
KSYEELXPSHNVYIM	KLEYNFNXPAKDPRIA

317 Linker1 primers	317 Linker2 primers
ELAKDPRxSHNVYIM	KLEYNFNxAATMENA
ELAKDPRxxSHNVYIM	KLEYNFNxxAATMENA
ELAKDPRxxxSHNVYIM	KLEYNFNxxxAATMENA

Fig. S6. Plot of ΔF/F for clarified lysate screen of cpGFP linker-screens at insertion points 165, 175, 311, and 317.

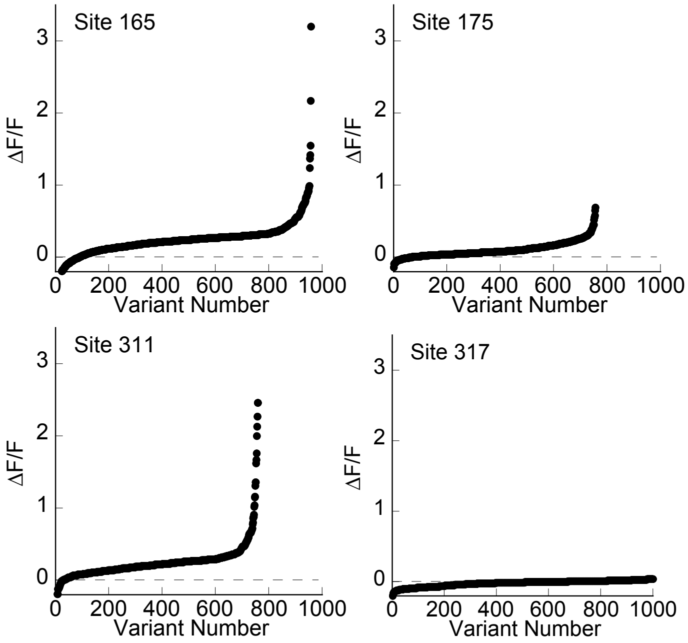

Fig. S7. MBP317-cpGFP ITC titration.

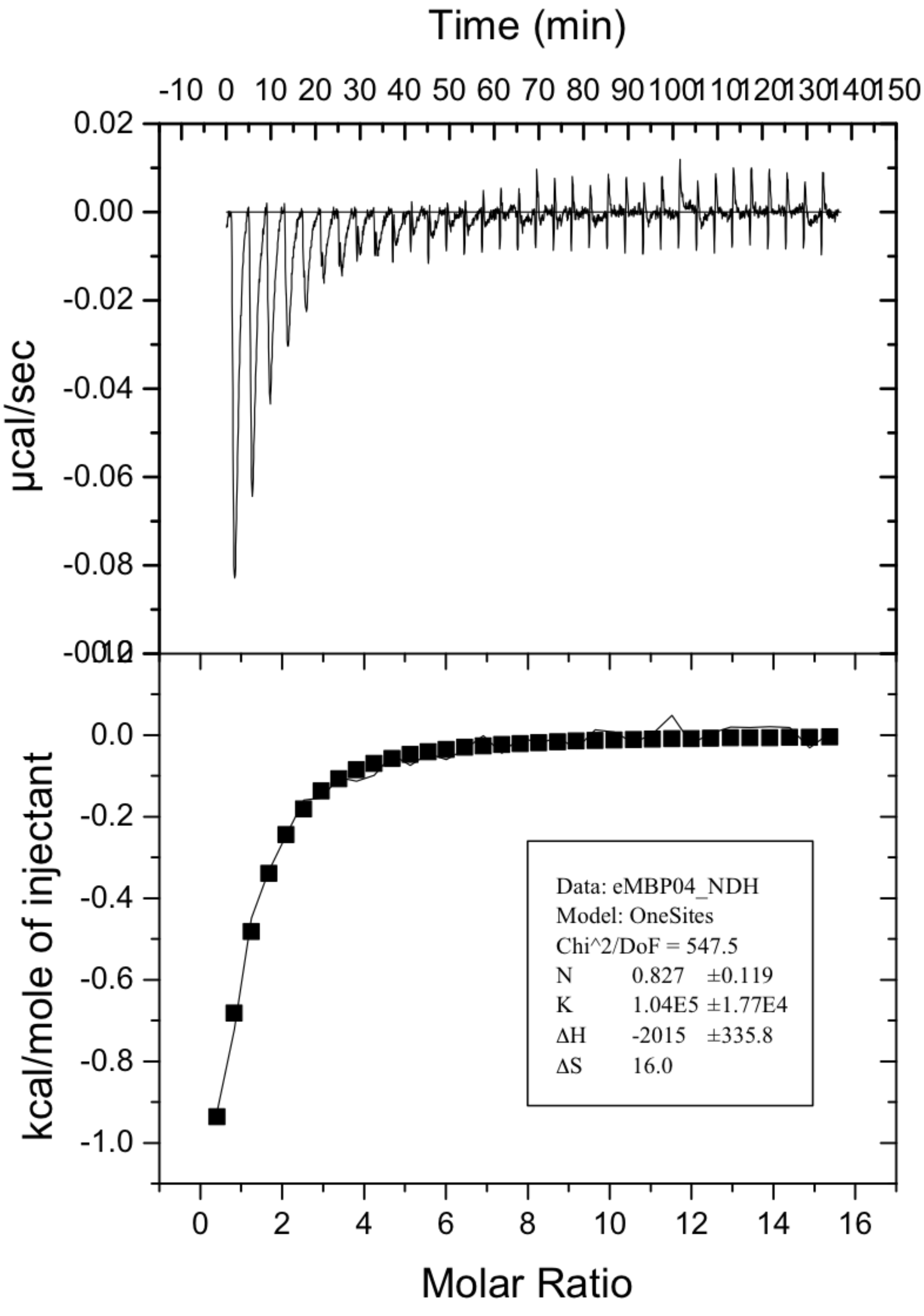

Fig. S8. Binding curves for affinity variants of MBP165-cpGFP.PPYF.

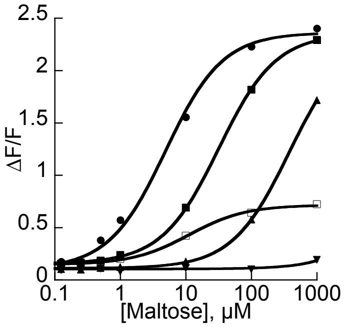

Fig. S9. Maltose and sucrose binding curves for wild-type and 5-7 variants of the MBP-cpGFP sensors.

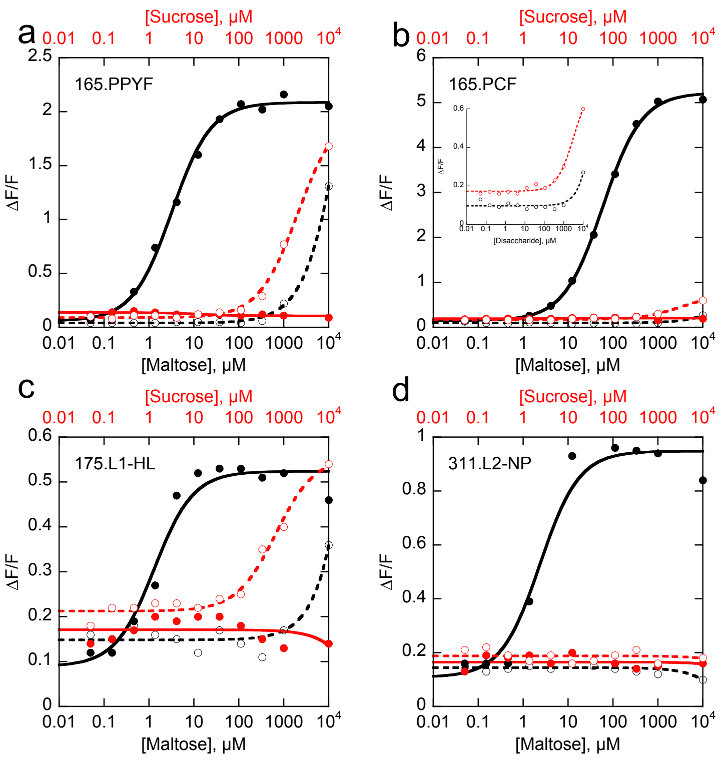

Fig. S10. Screening MBP165-cpBFP variants

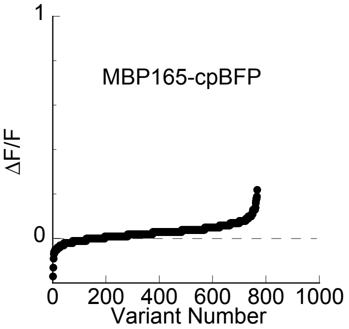

Fig. S11. *E. coli* images.

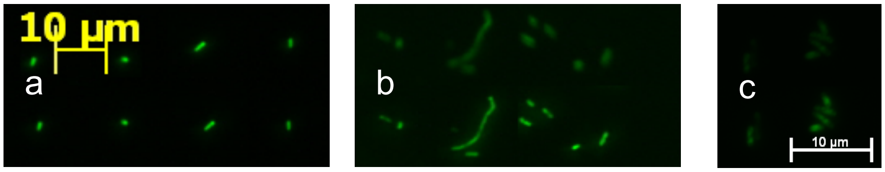

Fig. S12. 2-photon excitation spectra.

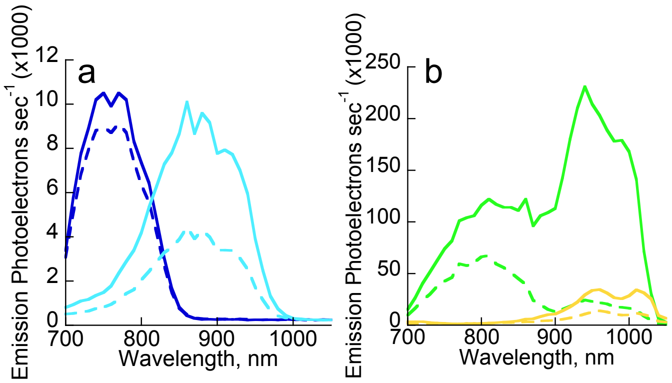

Fig. S13. Maltose concentration dependence of PPYF.T203V.

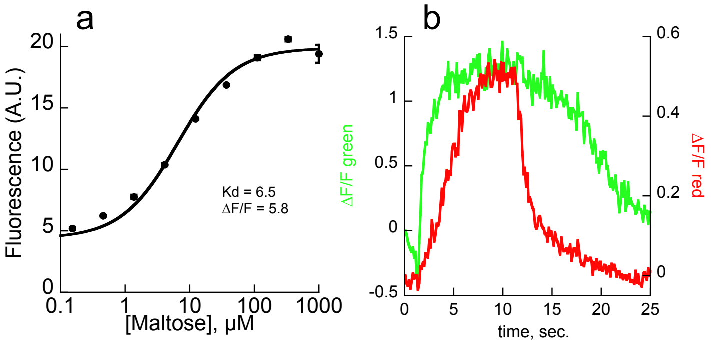

Fig. S14. Effect of GFP-T203V mutation.

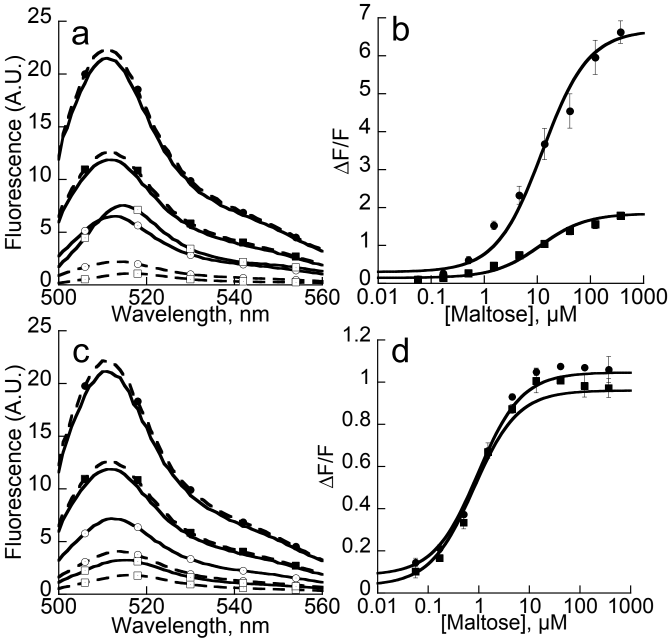

Supplementary Table S1. Peak fluorescence of individual bacteria.

EGFP			PPYF			PPYF. T203V		
Apo	Sat	ΔF/F	Apo	Sat	ΔF/F	Apo	Sat	ΔF/F
4095	3985	-0.03	2120	3266	0.54	553	1265	1.29
3840	3575	-0.07	943	2543	1.70	582	1405	1.41
3378	3244	-0.04	839	1260	0.50	600	1337	1.23
2384	2540	0.07	1226	2964	1.42	644	1328	1.06
1533	1547	0.01	1054	2557	1.43	583	1260	1.16
906	863	-0.05	1153	2359	1.05	613	1202	0.96
3542	3613	0.02	1025	2397	1.34	822	2028	1.47
			806	1763	1.19	555	1350	1.43
			756	1537	1.03	809	1844	1.28
			576	1084	0.88	580	1520	1.62

Supplementary Table S2. X-ray data collection and refinement statistics.

	MBP175-cpGFP.L1-HL	MBP311-cpGFP.L2-NP
**Data collection**		
X-ray beamline	APS 31-ID	APS 31-ID
Wavelength (Å)	0.9793	0.9793
Space group	$P2_12_12_1$	$P2_1$
Unit cell dimensions		
a, b, c (Å)	81.3, 88.7, 119.4	56.8, 74.8, 171.5
α, β, γ (°)	90, 90, 90	90, 97.5, 90
Resolution (Å)	31.41-1.90 (2.00-1.90)	34.24-2.00 (2.11-2.00)
R_{sym}	0.096 (0.592)	0.118 (0.530)
I / σI	14.3 (3.9)	11.4 (3.8)
Completeness (%)	99.9 (100)	100 (100)
Redundancy	11.1 (11.1)	5.7 (5.7)
**Refinement**		
Resolution (Å)	1.90	2.00
Number of reflections	65035	96467
R_{work} / R_{free} (%)	16.5/19.9	18.3/22.6
Number of atoms	5130	10102
Protein/maltose/water/sulfate	4803/23/294/10	9497/46/559/0
B-factors (Å^2)	19.5	20.6
Protein/maltose/water/sulfate	19.2/21.4/23.6/45.2	20.4/12.3/24.4/NA
RMSD values		
Bonds (Å)/angles (°)	0.027/2.019	0.022/1.868
Ramachandran plot (%)		
Favored/allowed/disallowed	98.3/1.7/0	98.1/1.7/0.2

Supplementary Information

Supplementary Figure Legends

Fig. S1-S4. Amino acid sequences of the framework MBP-cpGFP proteins and optimized variants. Linker sequences that were mutated are identified with black squares.

Fig. S5. Translation of the primers used for linker optimization of the MBP-cpGFP sensors. "x" indicates that a degenerate primer (with DNA sequence "NNS") was used to encode all 20 possible amino acids.

Fig. S6. Plot of ΔF/F for clarified lysate screen of cpGFP linker-screens at insertion points 165, 175, 311, and 317. The horizontal dashed line at zero indicates no fluorescence change. No error bars are plotted, but standard deviations in ΔF/F are less than 10% of an average ΔF. (Ex. 3 repetitions for MBP165-cpGFP.PPYF yields ΔF/F values of 2.51, 2.63, 2.54.) Artifacts resulting from incomplete clearing of lysate are easily resolved by a duplicate assay.

Fig. S7. ITC of MBP317-cpGFP demonstrates that the protein binds maltose, even though the linker screen failed to produce variants with a change in fluorescence.

Fig. S8. Binding curves for affinity variants of MBP165-cpGFP.PPYF. Data is fit to a single-binding site isotherm. Curve-fit affinities are: WT binding pocket, 5 μM (●); W230A, 32 μM (▪); W62A, 375 μM (▲); W340A , > 1mM (▼); I329W, 11 μM (□).

Fig. S9. Maltose (black) and sucrose (red) binding curves for wild-type (filled, solid lines) and 5-7 variants (open, dashed lines) of the MBP-cpGFP sensors. MBP165-

cpGFP.PPYF (a); MBP165-cpGFP.PCF (b); MBP175-cpGFP.L1-HL (c); MBP311-cpGFP.L2-NP (d).

Fig. S10. Plot of ΔF/F for clarified lysate screen of MBP165-cpBFP linker-screen. The horizontal dashed line at zero indicates no fluorescence change.

Fig. S11. Images of individual *E. coli* BL21(DE3) bacteria expressing (a) EGFP, (b) PPYF, or (c) PPYF.T203V in the absence (top) and presence (bottom) of maltose.

Fig. S12. 2-photon excitation spectra. MBP165-cpAzurite.L2-FE (a), –cpCFP.PCF (a), -cpGFP.PPYF (b), and –cpYFP.PPYF (b) were excited at the wavelengths indicated and emission measured through appropriate wavelength filters. Two graphs are shown to present different y-axis scales. Optimal ΔF/F values for 2-photon excitation of the spectral variants of MBP165 are: -cpAzurite, 1.1 (ex 760 nm); -cpCFP, 2.3 (ex 830-960 nm); -cpGFP, 10.0 (ex 940 nm); -cpYFP, 2.6 (ex 940 nm).

Fig. S13. Quantification of MBP165-cpGFP.PPYF.T203V fluorescence when displayed on the surface of HEK cells. (a) Concentration dependence. (b) Observed fluorescence after a “puff” of HBSS solution containing 1 mM maltose and 2.5 nM Alexa Fluor® 568 (Invitrogen, Carlsbad, CA). The onset of increasing green (maltose sensing) fluorescence and red (Alexa Fluor® 568) fluorescence is occurs at the same time, indicating that the sensor responds as quickly a diffusion in this set up. The prolonged response of the maltose sensor is likely a consequence of its high affinity for maltose, while the Alexa dye diffuses rapidly.

Fig. S14. Effect of GFP-T203V mutation. (a) Emission spectra of 1 μM purified eGFP (filled circles), cpGFP (filled squares), MBP165-cpGFP.PPYF (open circles), and

MBP165-cpGFP.PPYF+T203V (open squares) in the absence (dashed lines) or presence (solid lines) of 1 mM maltose. cpGFP is half as bright as eGFP, and the saturated MBP165-cpGFP.PPYF variants are about half as bright as cpGFP. Note that mutation T203V decreases the fluorescence of the apo-state of MBP165-cpGFP.PPYF and slightly increases the saturated state. (b) Titration of maltose for MBP165-cpGFP.PPYF (filled squares), and MBP165-cpGFP.PPYF+T203V (filled circles). Affinities for each protein are the same, but with different ΔF/F. (c) Emission spectra of 1 μM purified eGFP (filled circles), cpGFP (filled squares), MBP311-cpGFP.L2-NP (open circles), and MBP311-cpGFP.L2-NP+T203V (open squares) in the absence (dashed lines) or presence (solid lines) of 1 mM maltose. Note that mutation T203V decreases the fluorescence of both the apo-state and the saturated state of MBP311-cpGFP.L2-NP. (d) Titration of maltose for MBP311-cpGFP.L2-NP (filled squares), and MBP311-cpGFP.L2-NP+T203V (filled circles). Affinities for each protein are the same, but with ΔF/F slightly increased for the T203V variant.

Supplementary Table Legends

Table S1. Peak fluorescence of individual bacteria. MBP165-cpGFP.PPYF: Avg. ΔF/F = 1.11 ± 0.39; EGFP: Avg. ΔF/F = -0.01 ± 0.05.

Table S2. X-ray data collection and refinement statistics. MBP175-cpGFP.L1-HL bound to maltose was crystallized using the vapor diffusion method by mixing 1 µL of protein solution (10 mg/mL in 20 mM Tris pH 8.0, 100 mM NaCl, 3 mM maltose) with 1 µL of precipitant solution (0.5 M Ammonium sulfate, 0.1M Sodium citrate tribasic

dihydrate pH 5.6, 1.0 M Lithium sulfate monohydrate). Crystals of MBP175-cpGFP.L1-HL formed after 3 weeks and were cryoprotected in the precipitant solution supplemented with 25% glycerol for data collection. MBP311-cpGFP.L2-NP bound to maltose was similarly crystallized using vapor diffusion by mixing 1 µL of protein solution (30 mg/mL in 20 mM Tris pH 8, 100 mM NaCl, 8 mM maltose) with 1.5 µL of precipitant solution (0.1 M Sodium acetate pH 4.6, 8% w/v polyethylene glycol 4,000) at 23 °C. Crystals grew after 2 days and were cryoprotected with 30% ethylene glycol. X-ray diffraction data were collected at 100 K at beamline 31-ID of the Advanced Photon Source. Diffraction data were reduced using Mosflm (1) and Scala (2). The structures were solved using Phaser (3) from within CCP4 (4) to carry out a molecular replacement search sequentially with the cpGFP domain of the previously published GCaMP structure (residues 62-302 of PDB ID 3EKH) and the structure of maltose-bound MBP (PDB ID 1ANF). One molecule of the MBP175-cpGFP.L1-HL sensor and two MBP311-cpGFP.L2-NP sensors were present in the asymmetric units of their respective crystals. The structures were iteratively rebuilt using Coot (4) and refined with Refmac5 (5), including riding hydrogens and TLS refinement in later cycles. The final models are of high quality with good stereochemistry and include most amino acids except the affinity tags, a few residues at the N- and C-termini, and the glycine-containing linkers connecting the two halves of the cpGFP domains.

Supplementary References

1. Leslie A (1992) Recent Changes to the MOSFLM Package for Processing Film and Image Plate Data. *Joint CCP4 + ESF-EAMCB Newsletter on Protein Crystallography*.

2. CCP4 (1994) The CCP4 suite: programs for protein crystallography. *Acta Crystallogr D Biol Crystallogr* 50:760-763.

3. McCoy AJ, et al. (2007) Phaser crystallographic software. *J App Crystallogr* 40:658-674.

4. Emsley P & Cowtan K (2004) Coot: model-building tools for molecular graphics. *Acta Crystallogr D Biol Crystallogr* 60:2126-2132.

5. Murshudov GN, Vagin AA, & Dodson EJ (1997) Refinement of macromolecular structures by the maximum-likelihood method. *Acta Crystallogr D Biol Crystallogr* 53:240-255.
